# Rifaximin Alleviates Endotoxemia with Decreased Serum Levels of Soluble CD163 and Mannose Receptor and Partial Modification of Gut Microbiota in Cirrhotic Patients

**DOI:** 10.3390/antibiotics9040145

**Published:** 2020-03-29

**Authors:** Kosuke Kaji, Soichiro Saikawa, Hiroaki Takaya, Yukihisa Fujinaga, Masanori Furukawa, Koh Kitagawa, Takahiro Ozutsumi, Daisuke Kaya, Yuki Tsuji, Yasuhiko Sawada, Hideto Kawaratani, Kei Moriya, Tadashi Namisaki, Takemi Akahane, Akira Mitoro, Hitoshi Yoshiji

**Affiliations:** Department of gastroenterology, Nara Medical University, Kashihara 634-8522, Japan; saikawa@naramed-u.ac.jp (S.S.); htky@naramed-u.ac.jp (H.T.); fujinaga@naramed-u.ac.jp (Y.F.); furukawa@naramed-u.ac.jp (M.F.); kitagawa@naramed-u.ac.jp (K.K.); ozutaka@naramed-u.ac.jp (T.O.); kayad@naramed-u.ac.jp (D.K.); tsujih@naramed-u.ac.jp (Y.T.); yasuhiko@naramed-u.ac.jp (Y.S.); kawara@naramed-u.ac.jp (H.K.); moriyak@naramed-u.ac.jp (K.M.); tadashin@naramed-u.ac.jp (T.N.); stakemi@naramed-u.ac.jp (T.A.); mitoroak@naramed-u.ac.jp (A.M.); yoshijih@naramed-u.ac.jp (H.Y.)

**Keywords:** cirrhosis, endotoxin, microbiome, rifaximin

## Abstract

Rifaximin is a poorly absorbable antibiotic against hepatic encephalopathy (HE). This observational study aimed to elucidate the effect of rifaximin on intestinal permeability and gut microbiota in patients with decompensated cirrhosis. Thirty patients with decompensated cirrhosis were assessed by ammonia level, neuropsychological testing, endotoxin activity (EA), and serum proinflammatory cytokines at baseline and after four weeks of rifaximin treatment (1200 mg/day). Intestinal permeability was indicated by serum soluble CD163 (sCD163), mannose receptor (sMR), and zonulin levels. To evaluate the gut microbiome, 16S ribosomal RNA gene sequencing was applied. Rifaximin ameliorated hyperammonemia and cognitive dysfunction, although it did not change the serum proinflammatory cytokine levels. It decreased EA levels as well as serum levels of sCD163 and sMR, but not zonulin, and both decreases in sCD163 and sMR showed positive correlations with EA decrease (ΔsCD163: Correlation coefficient (R) = 0.680, *p* = 0.023; ΔsMR: R = 0.613, *p* = 0.014, vs. ΔEA). Gut microbial analysis revealed that the richness and complexity of species were unchanged while the abundance of the *Streptococcus* genus was reduced after treatment with rifaximin. Collectively, rifaximin alleviated HE and endotoxemia with improved intestinal hyperpermeability in patients with decompensated cirrhosis, and this effect is partially involved in a gut microbial change.

## 1. Introduction

Hepatic encephalopathy (HE) includes a wide spectrum of neurological and psychiatric manifestations ranging from subclinical alterations to comas caused by acute or chronic liver failure and portosystemic shunting [1,2,3]. HE occurs due to a merger of distinct pathophysiological abnormalities that may include systemic inflammation, oxidative stress, impaired blood–brain barrier permeability, neurotoxins, and impaired cerebral energy metabolism [1,2,3]. It has been suggested that these mechanisms cause minimal HE (mHE), the earliest stage of HE diagnosed by deficits in electroencephalopathy and neuropsychometric testing, as well as the overt stages of HE [4,5].

Most cirrhotic patients show elevated plasma endotoxin levels, and aberrant endotoxemia is a key player to induce inflammatory responses in the nervous system during the development of HE. Clinical evidence suggests a positive correlation between serum endotoxin levels and the incidence of overt HE [6,7]. Endotoxin levels are also significantly higher in portal blood from cirrhotic patients, suggesting that endotoxins are excessively produced by bacterial overgrowth and intestinal absorption is augmented. Increased absorption of endotoxins is often associated with the concept of “leaky gut”, which is characterized by increased intestinal permeability with defects in intestinal tight junction proteins (TJPs) [8]. In fact, TJP expression is reported to be inversely correlated with endotoxin levels [9]. This indicates that downregulation of intestinal TJPs leads to gut hyperpermeability and aberrant endotoxemia in cirrhotic patients.

Rifaximin has been extensively identified as a non-systemic, broad-spectrum, bactericidal antibiotic, and it proposed to bring about beneficial outcomes for patients with HE. Reportedly, rifaximin has been also suggested to improve endotoxemia, however there remains a long-standing debate about its underlying mechanisms. Although this drug is a non-absorbable antibiotic, several metagenomic profiles have revealed major compositional changes in the fecal microbiota after treatment [10,11,12]. Bajaj et al. noted that rifaximin could induce a shift from pathogenic to beneficial gut bacterial linkages with metabolites [10]. Moreover, Zhang et al. suggested the possible involvement of altered small-intestinal bacterial overgrowth (SIBO) in the effect of rifaximin [13]. However, it remains unclear whether the effect of rifaximin on endotoxemia is linked to its anti-leaky gut activity in cirrhotic patients with HE.

The purpose of this study is to assess the efficacy of rifaximin on intestinal permeability as well as gut microbiota in conjunction with improvement of endotoxemia in patients with decompensated cirrhosis. To non-invasively evaluate gut permeability, we employed three serum markers, namely soluble CD163 (sCD163), soluble mannose receptor (sMR), and zonulin. Both sCD163 and sMR are detectable as markers of macrophage activation in peripheral blood and associated with the severity of chronic liver diseases [14,15]. CD163, the hemoglobin–haptoglobin scavenger receptor, is expressed on macrophages and monocytes and is released into circulation as a soluble form (sCD163) [16]. Serum levels of sCD163 are highly correlated with the lipopolysaccharide (LPS)-related pathway in patients with alcoholic hepatitis [17]. The mannose receptor (MR) is also located on macrophages and dendritic cells, while sMR, the shedding product of MR, is increased in patients with liver diseases [14,18]. Intriguingly, a recent prospective study demonstrated that these markers are significantly correlated with the lactulose/mannitol ratio (LMR), a traditional parameter of intestinal permeability [19]. Circulating zonulin concentrations are also commonly used to assess intestinal permeability [20,21]. Zonulin is a protein that reversibly controls intestinal permeability by controlling binding between epithelial cells of the intestinal mucosa. Zonulin is involved in innate intestinal immunity and its concentration is also strongly correlated with LMR [22]. Lastly, we carried out metagenomic analysis by 16S ribosomal RNA (rRNA) gene sequencing to investigate alterations to the fecal microbiota.

## 2. Results

### 2.1. Patient Characteristics

**Figure 1:**
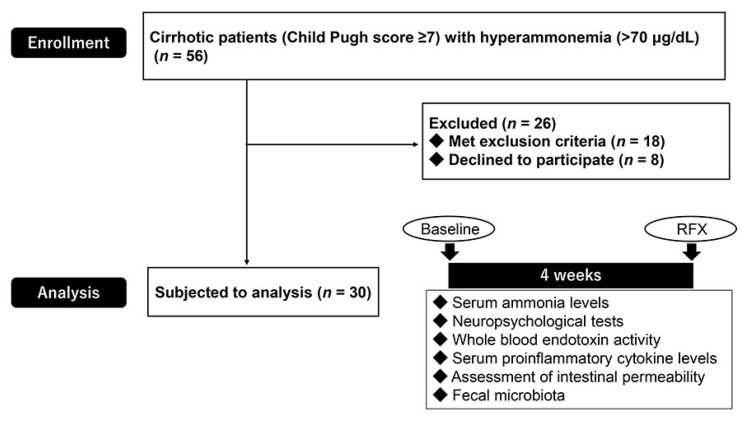
The selection of the study population and experimental design. A final number of 30 patients (after excluding 26 patients who met the exclusion criteria or declined to participate) were analyzed.

The trial flow chart is presented in Figure 1. Eighteen patients met the exclusion criteria and eight declined to participate, resulting in a cohort of 30 patients. The characteristics and demographics of the patients at pre-treatment (baseline) and post-treatment with rifaximin (RFX) are summarized in Table 1. This study included thirty patients with decompensated cirrhosis (median age: 67.3 (23–89) years). Four-week rifaximin treatment has no adverse effects, including digestive symptoms or renal dysfunction, during the study period. No statistical differences were shown in functional hepatic reserve, transaminases, blood counts and branched chain amino acid and tyrosine ratio (BTR) between baseline and after treatment with rifaximin (Table 1).

### 2.2. Rifaximin Improves Minimal Hepatic Encephalopathy with Reduced Endotoxin Activity

**Figure 2:**
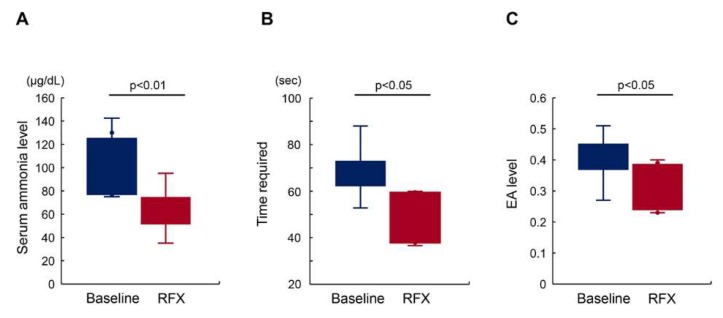
Effect of rifaximin on serum ammonia level, cognitive disturbance, and endotoxin activity (EA). (**A**) The mean serum ammonia levels among the patients who revealed high levels (>70 μg/dL) at baseline were significantly lowered 4 weeks after treatment with rifaximin (RFX). (**B**) Corresponding to serum ammonia levels, the mean time required for Number connection test-A (NCT-A) among patients who revealed prolongation (>50 s) at baseline was significantly shortened after treatment. (**C**) The mean endotoxin activity among patients who reported high levels (>0.4) at baseline significantly declined after treatment. Data are means ± SD.

At baseline, the number of patients with high endotoxin activity (EA) (>0.4) and delayed Number connection test-A (NCT-A) (>50 s) was 15 and 17, respectively. No patients experienced overt HE during the experimental period. Four-week rifaximin treatment significantly lowered the mean serum ammonia levels (101.9 ± 30.9 μg/dL at baseline vs. 63.3 ± 19.4 μg/dL at RFX; *p* < 0.01; Figure 2A). Corresponding to an improvement of hyperammonemia, the mean NCT-A time within the 17 patients who revealed prolongation (>50 s) at baseline was shortened by treatment with rifaximin (68.2 ± 9.6 s at baseline vs. 51.2 ± 14.0 s at RFX; *p* < 0.05; Figure 2B). In addition, the mean EA value within the 15 patients who showed high values (>0.4) at baseline declined after treatment (0.44 ± 0.06 at baseline vs. 0.32 ± 0.07 at RFX; *p* < 0.05; Figure 2C).

### 2.3. Rifaximin Lowers Serum sCD163 and sMR Levels

**Figure 3:**
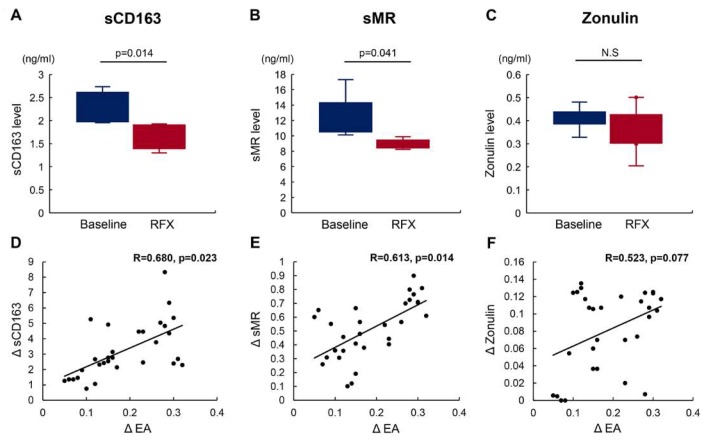
Effect of rifaximin on serum soluble mannose receptor (sMR), sCD163, and zonulin level. (**A**–**C**) Rifaximin decreased the serum levels of sCD163 (**A**) and sMR (**B**) but did not alter serum zonulin levels (**C**). (**D**–**F**) Univariate correlation analyses revealed that the decrease in the endotoxin activity level after treatment with rifaximin (ΔEA) positively correlated with the decreases in the serum sCD163 (**D**) and sMR levels (**E**). The decrease in the serum zonulin levels (Δzonulin) showed a marginally positive correlation with ΔEA (**F**). Data are means ± SD.

To evaluate the effects of rifaximin on leaky gut, we measured serum levels of intestinal permeability-associated markers including sCD163, sMR, and zonulin. Interestingly, serum levels of both sCD163 and sMR were markedly decreased by four-week rifaximin treatment (Figure 3A,B), while serum zonulin levels were unchanged (Figure 3C). Univariate correlation analyses revealed that the decline in EA value after treatment with rifaximin (ΔEA) was positively correlated with those in serum sCD163 and sMR levels (ΔsCD163: Correlation coeffient (R) = 0.680, *p* = 0.023; ΔsMR: R = 0.613, *p* = 0.014) (Figure 3D,E). The decline in serum zonulin level (ΔZonulin) showed a marginally positive correlation with ΔEA (R = 0.523, *p* = 0.077), but did not reach statistical significance (Figure 3F).

### 2.4. Alterations to Fecal Microbiota Composition

**Figure 4:**
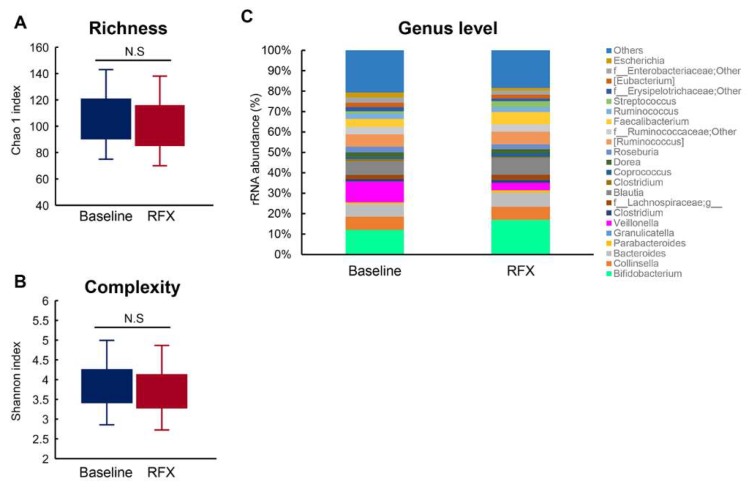
Fecal microbial analysis. (**A**) There was no statistically significant difference in the richness (Chao1 index) between the baseline and after rifaximin treatment groups. (**B**) There was no statistically significant difference in the complexity (Shannon index) between the baseline and after rifaximin treatment groups. (**C**) Taxonomic composition of fecal bacterial communities in the genus level. The relative abundance of *Veillonella* was decreased after 4 weeks of treatment with rifaximin, while the other genera were unchanged.

Fecal samples were collected from the 30 patients before and after rifaximin treatment and their gut microbiomes were analyzed using 16S rRNA gene sequencing. Between patients at baseline and those after treatment, there was no statistically significant difference in the richness (Chao1 index) (105.0 ± 38.5 at baseline vs. 92.1 ± 26.1 at RFX; *p* = 0.662; Figure 4A) and complexity (Shannon index) (3.857 ± 0.642 at baseline vs. 3.727 ± 0.591 after RFX treatment; *p* = 0.776; Figure 4B). Furthermore, 90 genera (58 Gram-negative and 32 Gram-positive) were detected in the fecal microbiome. Among the genera of Gram-negative bacteria, *Veillonella* decreased significantly after rifaximin treatment (*p* = 0.031) while the other genera, including Gram-negative bacteria such as *Bacteroides*, *Eubacterium*, and *Haemophilus*, remained largely unchanged (Figure 4C). Moreover, the proportion of Gram-positive bacteria were not altered by treatment with rifaximin (Figure 4C).

### 2.5. Rifaximin Does Not Affect Serum Proinflammatory Cytokine Levels

**Figure 5:**
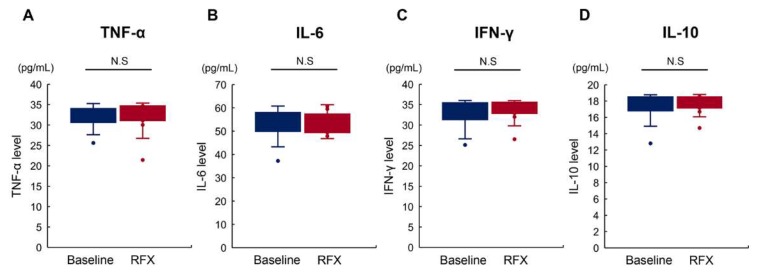
Effect of rifaximin in serum levels of proinflammatory cytokines. Rifaximin did not affect the serum levels of proinflammatory cytokines, including tumor necrosis factor (TNF)-α (**A**), interleukin (IL)-6 (**B**), interferon (IFN)-γ (**C**), and IL-10 (**D**).

Given the result that rifaximin reduced endotoxin activity, we then assessed changes in serum levels of proinflammatory cytokines, including tumor necrosis factor (TNF)-α, interleukin (IL)-6, interferon (IFN)-γ, and IL-10. As shown in Figure 5A–D, four-week administration of rifaximin did not affect the levels of these cytokines among patients showing a decrease in endotoxin activity following rifaximin treatment.

## 3. Discussion

As a highly specialized barrier against the passage of gut-derived antigens, the intestinal epithelium plays a pivotal role in gut immune homeostasis. The intestinal barrier can be structurally and functionally impaired by various factors, including bowel inflammation, excessive alcohol consumption, portal hypertension, and gut microbial changes [8,9,23,24,25]. Consequently, its permeability is aberrantly augmented and gut-derived bacteria (migration of microbes or their products into mesenteric lymph nodes), endotoxins, and pathogen-associated molecular patterns (PAMPs) translocate into enterohepatic circulation. It is well known that gut hyperpermeability is observed in patients with cirrhosis [25,26]. Assimakipoulos et al. demonstrated that TJPs, occludin, and claudin-1 are downregulated in duodenal biopsies from cirrhotic patients compared to healthy controls, and expression was inversely correlated with endotoxemia [27]. Several clinical studies have shown that increased intestinal permeability is closely associated with the severity of liver cirrhosis by the Child–Pugh classification and the pathogenesis of complications, including HE [28,29]. This study focused on the functional contribution of rifaximin on intestinal permeability in cirrhotic patients with HE. Our research has shown that other nonabsorbable antibiotics, polymyxins, and neomycins attenuate intestinal hyperpermeability by preventing the loss of TJPs in experimental cirrhotic models [30]. Moreover, a recent animal study demonstrated that rifaximin promoted the expression of occludin, the major TJP, in an irritable bowel syndrome mouse model [31]. Taken together, this evidence strongly suggests an interaction between improvement of HE and preservation of intestinal barrier function in rifaximin-treated patients.

We first demonstrated that a four-week treatment with rifaximin significantly lowered serum ammonia levels, improved cognitive performance, and decreased the EA value in the decompensated cirrhotic patients. The effects of rifaximin on plasma LPS level are controversial. For example, Finlin et al. reported that rifaximin treatment did not lower plasma LPS in obese humans [32]. Therefore, unlike other studies, we assessed the plasma endotoxin activity by an Endotoxin Activity Assay (EAA) which was separate from endotoxin concentration. Most quantitative limulus amebocyte lysate (LAL) tests, which are widely used to measure endotoxin levels, are not endotoxin specific, as these tests detect both endotoxins from Gram-negative bacteria and (1–3)-β-d-glucan from fungi, which are microbial products translocated from the intestine. Therefore, these tests find it difficult to detect spillover endotoxemia in liver diseases due to the complexity of the measurement, difficulty in standardization, and low sensitivity. The EAA is a novel and simple method to assess blood levels of endotoxins with higher sensitivity as compared with these tests [33]. Next, to verify our hypothesis, we evaluated alterations in intestinal permeability following rifaximin treatment in these patients. LMR is commonly used as a marker of hyperpermeability; the disaccharide lactulose is absorbed through the paracellular pathway (TJs) that corresponds to the permeability of large molecules, while the monosaccharide mannitol is absorbed by transcellular transport. This difference in intestinal absorption of two sugars indicates that LMR assessed by urinary excretion can reflect TJ-mediated intestinal permeability [34]. Although LMR is known to be markedly elevated in patients with advanced stage liver cirrhosis, LMR examination is invasive and time-consuming compared to simple serum collection. Furthermore, serum levels of sCD163, sMR, and zonulin have been proven to be positively correlated with LMR [14,15,16,17,18,19,20,21,22]. Thus, our study employed serum surrogate markers, namely sCD163, sMR, and zonulin, to non-invasively assess intestinal permeability. It was noteworthy that both sCD163 and sMR levels were significantly decreased by a four-week rifaximin treatment, and that these decreases showed highly positive correlations with improvements in endotoxemia, indicating that rifaximin might recover impaired intestinal permeability in cirrhotic patients. Unexpectedly, zonulin levels were not significantly altered by rifaximin, in spite of the changes in sCD163 and sMR. Zonulin levels are reported to relate to obesity and fatty liver [20]. In the present study, it was quite difficult to evaluate the presence of metabolic syndrome and histological liver steatosis, because the patients had already progressed to decompensated cirrhosis. Further large-scale analysis is required to assess the accuracy of zonulin levels.

In cirrhosis, gut microbial changes potently influence the pathogenesis of intestinal hyperpermeability [25,26]. Based on this evidence, we evaluated alterations in the fecal microbiota. Fecal metagenomic analysis revealed that treatment with rifaximin did not cause any significant changes in the overall richness and diversity. Analysis at the genus level showed significant decreases in the relative abundance of *Veillonella*. *Veillonella* is an intraorally indigenous bacterium that belongs to the anaerobic Gram-negative coccus [35]. Previous mucosal microbiome research has shown that *Veillonella* is more abundant in the colons of cirrhotic patients with HE compared to patients without HE [36]. The antimicrobial effect of rifaximin on *Veillonella* abundance has also been demonstrated in several clinical studies [11,12]. Remarkably, there were no changes in other Gram-negative bacteria before and after treatment with rifaximin. Moreover, although Gao et al. have proposed that rifaximin alters the relative abundance of *Lactobacillus* species in a rat model with stress-induced gut inflammation and visceral hyperalgesia, we did not identify a change in *Lactobacillus* after a four-week rifaximin treatment [37]. Therefore, we should further investigate whether the effect of rifaximin is dependent on the underlying disease and host species. The fecal microbial changes caused by rifaximin suggest a close relationship between reduced LPS production from *Veillonella* via suppressed overgrowth and improvement in leaky gut. However, this minor bacterial change appears insufficient to account for the effect of rifaximin on intestinal permeability.

TJ injuries involving intestinal barrier function are also associated with an endotoxin-stimulated systemic inflammatory response. Guo et al. reported that systemic inflammation caused an increase in intestinal permeability via an intracellular mechanism involving toll-like receptor 4 (TLR4)-dependent upregulation of CD14 membrane expression [38]. Therefore, we next investigated the impact of rifaximin on serum markers of systemic inflammation. The results revealed that rifaximin did not change serum levels of the proinflammatory cytokines TNFα, IL-6, IL-10, or IFN-γ, all of which are related to the LPS/TLR4 signaling pathway. This lack of an effect on inflammatory markers is consistent with the findings of a randomized trial comparing rifaximin with placebo for mHE [11]. These findings indicate that rifaximin affects the intestinal permeability independently of modified systemic inflammation, suggesting the involvement of another key mechanism.

Rifaximin is also known to pharmacologically function as an agonist of the pregnane X receptor (PXR), a nuclear receptor that senses the presence of toxic substances and drives detoxification [39]. Notably, a recent study demonstrated that microbial toxin-induced apoptosis and deprivation of TJPs in human intestinal cells were suppressed by rifaximin treatment through PXR-dependent inhibition of the TLR4/myeloid differentiation primary response 88 (MyD88)/nuclear factor kappa-light-chain-enhancer of activated B cells (NF-κB) pathway, indicating that rifaximin can directly improve impaired intestinal permeability [40]. This hypothesis could be tested by evaluating PXR activation in the intestinal epithelium of cirrhotic patients.

Our study has several limitations. First, it was performed as a single-center study with a small sample size. Further studies are needed to assess the effects of rifaximin on larger populations and to assess the effects of long-term administration. Second, since serum levels of sCD163 and sMR indirectly assess intestinal permeability, the results of LMR would be required for direct evaluation. Finally, a recent report from Kimer et al. claimed that rifaximin could not significantly affect the levels of sCD163 and sMR in decompensated cirrhotic patients [41]. Alteration in intestinal permeability is a common finding in patients with liver cirrhosis, although it has also been described in different entities associated with chronic liver disease such as alcohol-induced injury, non-alcohol fatty liver disease, and hepatitis C virus (HCV)-mediated injury [42]. Thus, we speculate the difference in the etiology of cirrhosis as a responsible factor to cause this discrepancy between both studies. Their findings conflict with our results, although this unique action of rifaximin also deserves to be further validated.

## 4. Methods

### 4.1. Subjects

The eligible patients attended Nara Medical University Hospital from January to December 2018 and were subject to this observational study. Initially, 56 patients diagnosed with decompensated cirrhosis (Child–Pugh score > 7) with hyperammonemia (>70 μg/dL) regardless of etiologies by means of clinical, biochemical, and imaging findings were enrolled. Major exclusion criteria were aged under 18 years; drank alcohol within 6 months prior to inclusion; refractory ascites; severe cardiac and/or respiratory dysfunction or renal failure (serum creatinine > 200 μmol/L); advanced cancer within the past 5 years; clinical or biochemical signs of infection a month prior to inclusion; concomitant inflammatory bowel disease and irritable bowel syndrome; previous history of gastrectomy, enterectomy, and/or liver transplantation; and developed portosystemic shunt which was detected by computed tomography. Patients who took non-absorbable disaccharides, probiotics or other antibiotics a month prior to inclusion were also excluded.

### 4.2. Study Design and Ethical Approval

All subjects were treated with 1200 mg of rifaximin per day for 4 weeks. The complete investigational program comprising measurements of serum ammonia, proinflammatory cytokines and intestinal permeability-associated marker levels, as well as an assessment of cognitive function, endotoxin activity (EA), and analysis of fecal microbiota, was performed at baseline and after the full rifaximin treatment course. Our protocols conformed to the ethical guidelines of the 1975 Declaration of Helsinki as reflected by the prior approval of the Ethics Committee of Nara Medical University Hospital (approval # 1637-2) and registered at University Hospital Medical Information Network (UMIN) 000032548. Informed consent was obtained from all individual participants included in the study.

### 4.3. Neuropsychological Testing

To clinically assess cognitive function, we employed the number connection test (NCT)-A. The software was developed by Otsuka Pharmaceutical Co., Ltd. (Tokyo, Japan), Kokuyo Co. Ltd. (Osaka, Japan), and IBS Japan Co., Ltd. (Tokyo, Japan), and distributed by The Japan Society of Hepatology. The hardware consists of a touch screen tablet such as an iPad (Apple Inc., Cupertino, CA, USA) [43]. The time required for NCT-A > 50 s was evaluated as abnormal cognitive function.

### 4.4. Whole Blood Endotoxin Activity

Measurement of whole blood EA is performed by using the endotoxin activity assay (EAA) kit (Spectral Diagnostics, Toronto, Canada), a chemiluminescent bio-assay based on the oxidative burst reaction of activated neutrophils to complement coated LPS–IgM immune complexes [33]. In brief, the EAA is based on the principle that endotoxins bind to anti-endotoxin antibodies and are delivered to neutrophils by complement receptors. In the presence of β-glucan and luminol, neutrophils undergo a respiratory burst accompanied by light emission. The light produced is quantified by a chemiluminometer, and its intensity is proportional to the amount of endotoxin present in the sample. An EA value > 0.4 was defined as abnormally high levels.

### 4.5. Proinflammatory Cytokines and Intestinal Permeability

The pro-inflammatory cytokines tumor necrosis factor-alpha (TNF-α), interleukin-6 (IL-6), interferon-gamma (IFN-γ), and interleukin-10 (IL-10) were measured using commercially available enzyme-linked immunosorbent assay (ELISA) kits. For non-invasive evaluation of intestinal permeability, serum levels of zonulin, sCD163, and soluble sMR were also measured using ELISA kits. TNF-α, IL-6, and IFN-γ were measured using kits from R&D Systems (Minneapolis, MN, USA), IL-10 was measured using Proteintech kit (Rosemont, IL, USA), zonulin was measured using a Immundiagnostik AG kit (Bensheim, Germany), sCD163 was measured using a CUSABIO kit (Houston, TX, USA), and sMR was measured using a LifeSpan BioSciences kit (Seattle, WA, USA). Assays were performed according to the manufacturer’s instructions.

### 4.6. Fecal Microbiome Analysis

Fecal samples were collected before and after a four-week rifaximin treatment and placed in 1.5 mL tubes, snap-frozen on dry ice, and stored at −80 °C. Using 16S rRNA analysis, the fecal samples were processed by Takara Bio (Shiga, Japan). DNA was extracted with a MoBio Powerlyzer Powersoil DNA isolation kit (MoBio Laboratories, Carlsbad, CA, USA). The V4 hypervariable region of the bacterial 16S rRNA gene was amplified from fecal DNA extracts using the modified universal bacterial primer pairs 341F (5′- TCGTCG GCAGCGTCAGATGTGTATAAGAGACAGCCTACGGGNGGCWGCAG-3′) and 806R (5′- GTCTCGTGGGCTCGGAGATGTGTATAAGAGACAGGGACTA CHVGGGTWTCTAAT-3′) with Illumina adapter overhang sequences. Amplicons were generated, cleaned, indexed, and sequenced according to the Illumina MiSeq 16S Metagenomic Sequencing Library Preparation protocol with slight modifications. Sequencing data were combined and sample identification was assigned to multiplexed reads using the MOTHUR software environment [44]. The data were denoized; low-quality sequences, pyrosequencing errors, and chimeras were removed, and the sequences were then clustered into operational taxonomic units (OTUs) at 97% identity using the CD-HITOTU pipeline [45]. OTUs containing fewer than four reads per individual diet/animal combination were excluded due to the likelihood of a sequencing artifact. The samples were normalized by random resampling sequences using the lowest number of sequences per sample (each diet/animal combination) using Daisychopper (http://www.festinalente.me/bioinf/). Taxonomic classification of OTUs was performed with the Ribosomal Database Project Classifier [46].

### 4.7. Statistical Analysis

The Mann–Whitney U test was used to analyze the differences between paired and unpaired groups. The Spearman rank test was applied to calculate interrelation. Data are indicated as means ± standard deviation (SD). Statistical significance was defined as a two-tailed *p*-value less than 0.05. The statistical software ‘EasyR’ (EZR), which is based on R and R commander, was used for analyses. EZR enables the application of statistical functions that are frequently available for clinical studies, such as survival analyses, including competing risk analyses and the use of time-dependent covariates, receiver operating characteristics analyses, meta-analyses, and sample size calculations [47].

## 5. Conclusions

Rifaximin treatment efficiently decreased endotoxin activity with improvement in intestinal hyperpermeability. This effect was associated with a minor change in bacterial composition, but no alterations to systemic inflammatory status. Further studies are needed to elucidate possible mechanisms that may protect intestinal barrier function.

## Figures and Tables

**Table 1:** Characteristic features of patients (*n* = 30).

Parameters	Baseline	RFX	*p* Value
Age		67.3 (23–89)	
Sex (male/female)		18/12	
Etiology			
Alcohol		5 (16.7%)	
Hepatitis B virus (HBV)		4 (13.3%)	
Hepatitis C virus (HCV)		11 (36.7%)	
Non-alcoholic steatohepatitis		4 (13.3%)	
Autoimmune hepatitis		2 (6.7%)	
Primary biliary cholangitis		2 (6.7%)	
Alcohol + HBV or HCV		2 (6.7%)	
Child class (A/B/C)		0/28/2	
MELD score	8.5 (1.3–17.4)	7.9 (1.7–18.5)	0.546
Child-pugh score	7 (7–13)	8 (7–12)	0.539
Aspartate aminotransferase (U/L)	47 ± 21	52 ± 30	0.507
Alanine aminotransferase (U/L)	31 ± 15	30 ± 15	0.928
Albumin (g/dL)	3.3 ± 0.6	3.4 ± 0.6	0.761
Total bilirubin (mg/dL)	1.7 ± 0.8	1.5 ± 0.7	0.412
Prothrombin time (INR)	1.30 ± 0.12	1.29 ± 0.13	0.819
C-reactive protein (mg/dL)	0.3 ± 0.5	0.3 ± 0.4	0.916
Leukocyte (10^3^/μL)	3.8 ± 1.9	3.8 ± 1.9	0.95
Platelet (10^4^/μL)	8.4 ± 3.8	8.2 ± 3.9	0.9
Branched chain amino acid and tyrosine ratio	3.6 ± 1.4	4.3 ± 3.5	0.33

RFX = rifaximin. Data are expressed as median and total range (age, Model for End-Stage Liver Disease [MELD] and Child–Pugh score), and as mean ± standard deviation (SD) (the others).

## Abbreviations

HE	hepatic encephalopathy
EA	endotoxin activity
sCD163	soluble CD163
sMR	mannose receptor
rRNA	ribosomal RNA
mHE	minimal hepatic encephalopathy
TJPs	tight junction proteins
SIBO	small-intestinal bacterial overgrowth
LPS	lipopolysaccharide
LMR	lactulose/mannitol ratio
NCT	number connection test
EAA	endotoxin activity assay
TNF-α	tumor necrosis factor-alpha
IL-6	interleukin-6
IFN-γ	interferon-gamma
IL-10	interleukin-10
ELISA	enzyme-linked immunosorbent assay
OTUs	operational taxonomic units
SD	standard deviation
MELD	median model of end-stage liver disease
CRP	C-reactive protein
WBC	white blood cells
BTR	branched chain amino acid and tyrosine ratio
PAMPs	pathogen-associated molecular patterns
TLR4	toll-like receptor 4
PXR	pregnane X receptor
HBV	hepatitis B virus
HCV	hepatitis C virus
NASH	non-alcoholic steatohepatitis;
AIH	autoimmune hepatitis
PBC	primary biliary cholangitis
